# Targeting the Protein Tunnels of the Urease Accessory Complex: A Theoretical Investigation

**DOI:** 10.3390/molecules25122911

**Published:** 2020-06-24

**Authors:** Matteo Masetti, Federico Falchi, Dario Gioia, Maurizio Recanatini, Stefano Ciurli, Francesco Musiani

**Affiliations:** 1 Laboratory of Computational Medicinal Chemistry, Department of Pharmacy and Biotechnology, University of Bologna, 40126 Bologna, Italy; matteo.masetti4@unibo.it (M.M.); federico.falchi@hotmail.com (F.F.); maurizio.recanatini@unibo.it (M.R.); 2 Computational and Chemical Biology, Istituto Italiano di Tecnologia, Via Morego 30, 16163 Genova, Italy; dario.gioia@iit.it; 3 Laboratory of Bioinorganic Chemistry, Department of Pharmacy and Biotechnology, University of Bologna, 40126 Bologna, Italy; stefano.ciurli@unibo.it

**Keywords:** urease, *Helicobacter pylori*, virtual screening, protein tunnels, protein–protein interaction

## Abstract

Urease is a nickel-containing enzyme that is essential for the survival of several and often deadly pathogenic bacterial strains, including *Helicobacter pylori*. Notwithstanding several attempts, the development of direct urease inhibitors without side effects for the human host remains, to date, elusive. The recently solved X-ray structure of the *Hp*UreDFG accessory complex involved in the activation of urease opens new perspectives for structure-based drug discovery. In particular, the quaternary assembly and the presence of internal tunnels for nickel translocation offer an intriguing possibility to target the *Hp*UreDFG complex in the search of indirect urease inhibitors. In this work, we adopted a theoretical framework to investigate such a hypothesis. Specifically, we searched for putative binding sites located at the protein–protein interfaces on the *Hp*UreDFG complex, and we challenged their druggability through structure-based virtual screening. We show that, by virtue of the presence of tunnels, some protein–protein interfaces on the *Hp*UreDFG complex are intrinsically well suited for hosting small molecules, and, as such, they possess good potential for future drug design endeavors.

## 1. Introduction

All living organisms require transition metal ions as indispensable micronutrients [1,2]. On the other hand, the low environmental availability coupled with their toxicity obliged all life forms to develop mechanisms for selective metal ion import, trafficking, accumulation, concentration regulation, and export [3,4,5,6,7]. Despite their biological and biophysical relevance, metal trafficking processes in the cell and inside proteins are still poorly understood [5,7], even though their relevance has been highlighted for the development of new therapeutics [8]. Here, we focus on the trafficking of Ni(II) ions, which are essential for the survival of several and often deadly pathogenic bacterial strains, such as *Helicobacter*, *Staphylococcus*, *Clostridium*, *Vibrio*, *Mycobacterium*, *Yersinia*, *Escherichia*, *Proteus*, *Ureaplasma*, *Klebsiella*, *Pseudomonas*, *Corynebacterium*, *Providencia*, *Morganella*, and *Cryptococcus*.

One of the common factors of these microorganisms is the nickel-dependent enzyme urease (urea amidohydrolase; EC 3.5.1.5), which is one of the main players in colonization and for survival in the host organisms [9,10,11]. Urease catalyzes urea hydrolysis to yield ammonia and bicarbonate, which, in turn, cause an increase in the local pH up to values suitable for bacterial survival. This is the case for *Helicobacter pylori* (*Hp*), a neutrophilic bacterium able to survive in the highly acidic gastric niche [12]. The Center for Disease Control and Prevention estimates that approximately two-thirds of the world’s population harbor *H. pylori*, whose infection can lead to stomach cancer and cause gastric mucosa-associated lymphoid tissue lymphoma [13]. Indeed, *H. pylori* is the only bacterium that has been classified as a class-I carcinogen in humans by the International Agency for Research on Cancer, and its importance has been emphasized by the award of the Nobel Prize in Medicine to Marshall and Warren in 2005, for their studies on the link between *H. pylori* infection and gastritis and peptic ulcers. Moreover, the rising antibiotic resistance that affects the most commonly used *H. pylori* eradication treatments requires the identification of new drug targets [14,15]. Indeed, eight out of twelve dangerous microorganisms recently identified by the World Health Organization for their antibiotic resistance use urease for their survival [16]. To this aim, if one considers that Ni(II) ions are not essential for higher animal species, nickel metabolism is an ideal candidate for the development of new specific therapeutics to tackle bacterial pathogens [17]. Indeed, it has been shown that nickel has some beneficial effects on the health of experimental animal models and that its deprivation induces detrimental effects on bone health, cGMP signal transduction, and carbohydrate and lipid metabolism, among others [18,19,20]. It has been suggested that nickel might affect the function of gaseous molecules, such as O_2_, CO_2_, CO, and NO [18,20]. On the other hand, the nutritional effect of nickel in humans has not yet been studied sufficiently [20].

**Figure 1:**
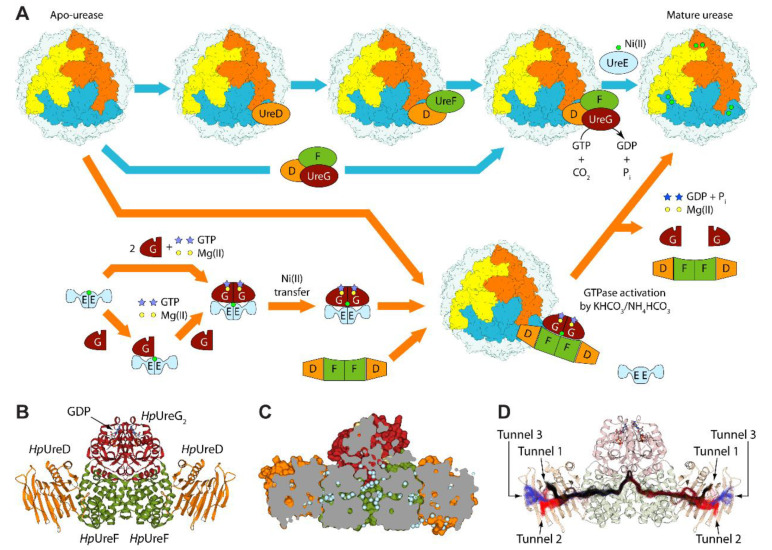
(**A**) Quaternary structure of urease from *H. pylori* (Protein Data Bank (PDB) id 1E9Z) and schematic representation of the proposed mechanisms for urease activation. The colored chains highlight the trimer that constitutes the minimal quaternary structure of urease, while the other three trimers constituting the active form of the enzyme in *H. pylori* are in grey. The Ni(II) ions (located at the bottom of the reaction site cavity) are shown as green circles. (**B**) Ribbon diagram and (**C**) longitudinal section of the solvent-excluded surface of the apo *Hp*UreDFG crystal structure (PDB id 4HI0). *Hp*UreD, *Hp*UreF, and *Hp*UreG chains are colored as in Panel (**A**). Water molecules are depicted as light blue spheres, and GDP is shown as balls-and-sticks and colored according to atom type. (**D**) Ribbon diagram of the *Hp*UreDFG complex and tunnels identified throughout the molecular dynamics (MD) simulation, all depicted in one frame as the tunnel centerlines. Tunnels 1, 2, and 3 are reported in black, blue, and red, respectively.

The crystal structures of ureases from several bacteria and higher plants available in the Protein Data Bank (PDB) reveal a nearly identical conserved quaternary structure constituted by a functional minimal trimeric assembly [9,10,11]. Each monomer of the latter is in turn composed of a single chain in ureases from higher plants [21,22], by two chains in the case of *H. pylori* [23], and by three chains in the cases of *Sporosarcina pasteurii* and *Klebsialla aerogenes* [9,10,11]. The minimal trimeric assembly can eventually dimerize in higher plants, while it generates nearly spherical tetramers in *H. pylori* [23] (Figure 1A). Each monomer of the minimal trimeric assembly hosts one conserved active site containing two Ni(II) ions bridged to a carbamylated lysine residue [9,10,11]. Urease inhibition has been the subject of several studies [11,24,25,26,27,28,29,30,31,32,33,34,35,36]. Unfortunately, to date, it has not been possible to develop a molecule that is not toxic for human health [37]. Thus, instead of focusing on the mature enzyme, here, we focus on the urease activation mechanism that leads from the inactive apo-urease (synthesized in vivo in an inactive form devoid of the Ni(II) ions and without any modification on the active site’s lysine residue) to its active holo-form.

The activation of the enzyme requires the action of four accessory proteins, named UreD (called UreH uniquely in *H. pylori*), UreF, UreG, and UreE, together with guanosine-5’-triphosphate (GTP) hydrolysis and CO_2_ uptake [9,11]. According to the first activation mechanism first proposed in 1997 [38] and modified in the subsequent years (Figure 1A), UreD appears to be the first protein that binds apo-urease, while UreF is proposed to bind the apo-urease:UreD complex through direct interaction with UreD. UreF is then allegedly able to facilitate the formation of the complex between apo-urease and the UreD:UreF:UreG complex (UreDFG hereafter) [39]. UreG is a small GTPase proposed to couple GTP hydrolysis to the process of urease activation and is the first reported case of an intrinsically disordered enzyme [40], possessing disordered regions involved in the recognition of other ureases’ accessory proteins [41]. UreF has also been proposed to act as a GTPase-activating protein (GAP) to regulate the function of UreG [42]. Finally, UreE is a metallo-chaperone deputed to the delivery of Ni(II) ions to the apo-urease:UreDFG complex in a GTP-dependent activation process [43]. A variant of this activation mechanism involves the direct interaction of a preformed UreDFG protein complex with the inactive form of the enzyme [9]. However, a more recent proposal suggests a different order of events (Figure 1A): (i) first, a Ni(II) ion is transferred from UreE to UreG when the latter is not yet bound to the UreF and UreD accessory proteins [44,45,46]; (ii) then, the Ni(II)-loaded UreG separates from UreE to bind the preformed UreD:UreF assembly and form the Ni(II)-bound UreDFG complex [46]; (iii) the latter finally interacts with apo-urease and allows the insertion of two Ni(II) ions into the enzyme in a process that requires GTP hydrolysis and activation by bicarbonate [46]. 

The crystal structures of (*Hp*UreF)_2_ [47], (*Hp*UreDF)_2_ [48], and (*Hp*UreDFG)_2_ [49] from *H. pylori* devoid of metal ions provided a structural framework for understanding the process of Ni(II) ion delivery to the apo-urease active site. The *Hp*UreDFG structure (Figure 1B) features a central core composed of *Hp*UreF flanked by one monomeric chain of *Hp*UreD bound to each side of the *Hp*UreF dimer, while the *Hp*UreG dimer is bound to a large concave region formed on the surface of the *Hp*UreF homodimer. In the *Hp*UreDFG supercomplex, a GDP molecule is bound to each *Hp*UreG monomer. An in depth analysis of the *Hp*UreDFG structure, coupled with results from site-directed mutagenesis, resulted in the discovery of a large cavity at the interface between *Hp*UreF and *Hp*UreG, starting from the UreG loop containing a conserved Cys–Pro–His (CPH) motif proposed to bind nickel ions [50] (Figure 1C). Moreover, it has been shown that the cavity contains several water molecules interconnected through a network aligned along the long horizontal axis of the UreD–UreF_2_−UreD portion of the complex [50]. This led to the identification of two nearly identical and symmetric tunnels starting from the central cavity in the complex and exiting near the *Hp*UreD C-terminal, passing through *Hp*UreF. The original hypothesis that the tunnels of the *Hp*UreDFG complex are involved in the transport of nickel ions received further support from in vivo assays using site-directed mutagenesis coupled with bioinformatics and atomistic molecular dynamics (MD) simulations on UreD from *K. aerogenes* (*Ka*UreD) [51]. A more recent study involving atomistic MD simulations run on the entire *Hp*UreDFG complex (Figure 1D) showed that the tunnels observed in the *Hp*UreDFG crystal structure are stable in solution at the hundreds of nanoseconds time scale [52]. The same study identified three possible tunnel exits on the surface of *Hp*UreD [52].

Here, we investigate the possibility of hampering the maturation of the urease system by targeting the functionality of the *Hp*UreDFG protein complex. Relying on theoretical arguments, we show that small molecules identified through a structure-based virtual screening (SBVS) approach might act as indirect urease inhibitors by either disrupting protein–protein interactions within the *Hp*UreDFG complex or blocking the Ni(II) transfer. Our findings are complemented by MD simulations carried out on the docked complexes to assess the structural and energetic reliability of the predicted binding modes. We show that specific regions of the *Hp*UreF protein are intrinsically suited to accommodating small molecule binding and might hold significant potential for future drug discovery endeavors.

## 2. Results and Discussion

### 2.1. Identification of Druggable Pockets

**Figure 2:**
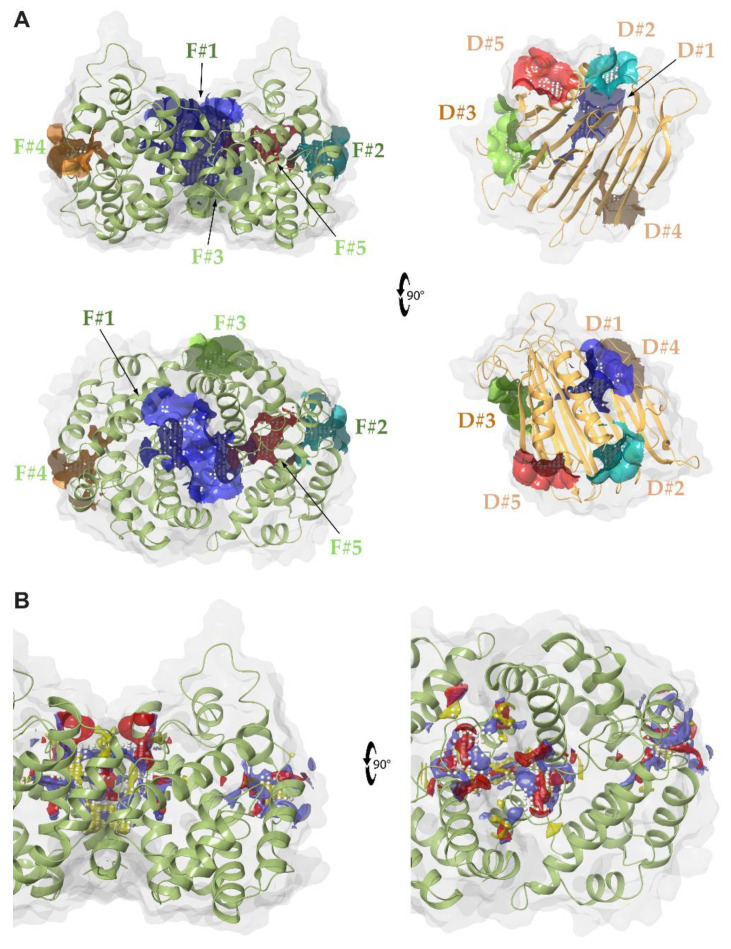
(**A**) Main sites identified by SiteMap on the *Hp*(UreF)_2_ and *Hp*UreD structures. For both proteins, the molecular surfaces of the druggable Sites #1, #2, #3, #4, and #5 are colored in blue, cyan, green, orange, and red, respectively, while the remaining parts of the surfaces are in white. The proteins’ ribbons are colored as in Figure 1. The proteins in the bottom panels have been rotated by 90° with respect to the orientations reported in the top panels. (**B**) Details of Sites F#1 and F#2, reporting the results of the SiteMap analysis. White dots represent the grid where the placement of ligand atoms is allowed in the SiteMap analysis. Hydrogen bond donors and acceptors are represented by red and blue surfaces, respectively, while the hydrophobic surfaces are in yellow. The protein in the right panel has been rotated by 90° with respect to the orientations in the left panels.

The *Hp*UreDFG protein assembly is a complex structure with a surface of ca. 49,000 Å^2^. It also hosts an intricate network of tunnels that are supposed to translocate Ni(II) ions from the CPH motif of (UreG)_2_ into the active site of urease [50,52] (Figure 1). In order to identify possible binding sites for small molecules, the whole complex was decomposed into its main building blocks, namely the (UreG)_2_ and (UreF)_2_ dimers and the two UreD monomers (see Figure 2A). The rationale was to exploit the openings of the tunnels at the interface region of the aforementioned building blocks as potential sites for disrupting protein–protein interactions (PPIs). The SiteMap tool available in the Schrödinger suite was used for this aim. In particular, SiteMap was employed on the whole (UreF)_2_ dimer, while for symmetry reasons, only a single UreD monomer was taken into account. On the contrary, (UreG)_2_ was not considered at all in the analysis. Indeed, (UreG)_2_ shows a quite convex surface at the (UreF)_2_:(UreG)_2_ interface and is known to be an intrinsically disordered protein able to fold upon interaction with the correct partner [40], making it unsuitable for virtual screening. Moreover, the tunnels for Ni(II) translocations are expected to originate from the CPH motif found at the interface between the (UreG)_2_ and (UreF)_2_ dimers. From this standpoint, the larger concave surface of (UreF)_2_ and the presence of tunnels in the assembled complex (see Figure 1D) led us to focus on this dimer and exclude (UreG)_2_ from further analysis.

As Figure 1B–D show, each UreD monomer binds the (UreF)_2_ dimer at the longitudinal sides of the complex. The symmetrical internal tunnel departing from the CPH motif at the (UreF)_2_:(UreG)_2_ interface splits up at almost the center of the complex, giving rise to two branches penetrating through each UreF monomer towards the UreD monomers. Each tunnel then proceeds along UreD, where it further trifurcates, reaching the protein surface through three different pathways (labeled as Tunnel 1, Tunnel 2, and Tunnel 3 in Figure 1D). For both UreD and UreF, up to five sites were allowed to be identified by SiteMap. If one considers the binding sites on the UreD surface, Sites D#1 and D#4 are located at the exits of Tunnels 1 and 2. Indeed, the exit of Tunnel 2 is where the (UreDFG)_2_ complex has been proposed to bind apo-urease [51]. Conversely, Sites D#2 and D#5 are located on the upper side of the protein in the vicinity of the interface between the α-helices and β-sheet core of UreD. Site D#3 is found at the UreD:(UreF)_2_ interface, at the entrance of the UreF:UreD tunnel on the UreD side. On the (UreF)_2_ dimer, we note that Site F#1 represents the cavity found at the (UreF)_2_:(UreG)_2_ interface where the tunnels originate. This is also the largest pocket among those identified by SiteMap (see Table 1). Sites F#2 and F#4 are symmetrically located at each of the UreD:(UreF)_2_ interfaces, while F#3 is found on the back side of the complex (according to the orientation reported in Figure 1B–D), in the vicinity of the interface between the two UreF monomers. Conversely, Site F#5 is an internal pocket communicating with both F#1 and F#2. Taken as a whole, Sites F#1, F#5, and F#2 run along the internal tunnel crossing UreF and leading to UreD. Moreover, as Figure 2A shows, D#3 and F#2 turned out to be complementary sites, as they face the internal tunnel that crosses the two proteins at the same interface region. The lack of symmetric counterparts for D#3 and D#5 is probably due to the limit of five detectable sites as defined by the default SiteMap settings.

The SiteScore and the DrugScore resulting from the SiteMap analysis are reported in Table 1. The SiteScore employs size, enclosure, and hydrophilic terms to characterize a protein patch, while the DrugScore uses the same parameters with different coefficients. In particular, a SiteScore ≥ 0.80 is useful for identifying sites that can potentially bind ligands based on the dataset originally employed to train the model [53,54]. On the other hand, a druggable binding site is expected to show a DrugScore ≥ 0.98, while values lower than 0.83 identify undruggable binding sites [53,54]. A SiteScore value equal to or larger than 0.80 was determined for Sites D#1 and D#2 on the UreD monomer and for all the identified sites of the (UreF)_2_ dimer except for Site F#3. On the other hand, only Sites D#1 and F#1 resulted in a DrugScore larger than 0.98, all the others having values smaller than 0.83. According to this classification, therefore, only Sites D#1 and F#1 were considered to display high druggability. However, while F#1 is associated with a well characterized interface (i.e., the (UreF)_2_:(UreG)_2_ interface), D#1 is not, as it has been characterized only through indirect observations [51]. This suggests that only F#1 can be safely exploited in the search for drugs for disrupting PPIs. However, even though F#2 cannot be strictly classified as a druggable site (DrugScore = 0.82), we chose to consider it for further analysis because of its borderline behavior with respect to the druggability threshold of 0.83.

In summary, this analysis led us to the identification of potentially druggable binding sites located on the surface of the (UreF)_2_ dimer, namely Sites F#1 and F#2. Notably, these sites are highly dissimilar in terms of both volume and shape (see Table 1 and Figure 2B, respectively). Moreover, while Site F#1 is located at the (UreF)_2_:(UreG)_2_ interface, the UreD:(UreF)_2_ side is involved in the case of F#2. Thus, from the standpoint of PPI disruption, targeting these sites would lead to hampering the assembly of the *Hp*(UreDFG)_2_ complex with distinct mechanisms. Since the molecular features encoded by these sites are different, it is reasonable to expect that they are also differently equipped for interacting with potential PPI disruptors.

### 2.2. Challenging the (UreF)_2_ Druggable Sites through Virtual Screening

Having identified the more suitable binding sites for interacting with potential PPI disruptors on the surface of the (UreF)_2_ dimer, it is necessary to investigate their propensity to bind drug-like molecules in a greater level of detail. Here, we exploited an SBVS approach to challenge the ability of Sites F#1 and F#2 to bind potential inhibitors of the urease activation system. Specifically, an SBVS was performed using a database downloaded from the ZINC website [55] and properly prepared as reported in the Methods section for each of the previously identified sites. The top-ranked molecules of each site were visually inspected, and to help the rationalization of the molecular properties shared by potential binders, the SBVS outcome was further processed (see Methods).

As a general trend, we observed that molecules resulting from the SBVS performed on the F#1 site showed much better docking scores than those obtained from Site F#2 (the docking scores ranged from −10.88 to −8.81 for F#1, and from −8.26 to −6.00 and F#2). This behavior was not entirely unexpected, as Site F#1 is much larger than F#2 (compare the volume of the sites in Table 1), thus offering more residues for establishing potentially favorable interactions with small molecules (even though fragments can show a high ligand efficiency in small sites). Notably, the larger volume of Site F#1 also reflects the fact that it is found at the top-side of the (UreDFG)_2_ dimer and near the UreF:UreF interface, in the binding region of the (UreG)_2_ dimer. As such, F#1 is partly made by the entrance of the two tunnels that are traveling towards the lateral sides of the *Hp*(UreDFG)_2_ complex in opposing directions (see Figure 1D). As we will see later, the C_2_ symmetry of F#1 has also a non-negligible impact on the chemical structures prioritized by the SBVS.

**Figure 3:**
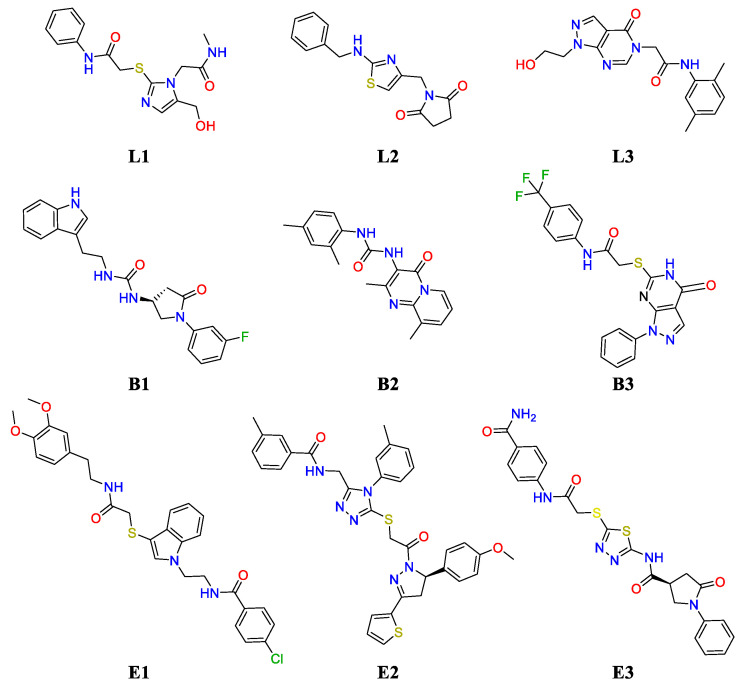
Molecular structure of the prioritized molecules obtained from the structure-based virtual screening (SBVS).

**Figure 4:**
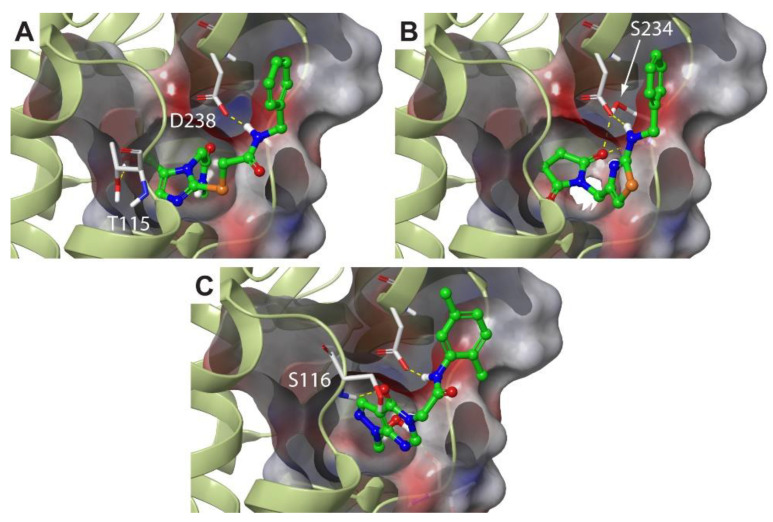
Docking poses for Compounds **L1–3** (Panels **A**–**C**, respectively) in the F#2 site. *Hp*(UreF)_2_ ribbons are in dark green, and residues involved in the binding are labeled and depicted as thin sticks colored according to atom type. The **L1–3** compounds are shown as green sticks, where heteroatoms are colored according to atom type. Hydrogen bonds are shown using yellow dashed lines.

Concerning the SBVS outcome for the F#2 site, we found that the top-ranked binders were mostly fragment-like molecules with molecular weights (MWs) lower than 300. This behavior can be easily explained considering the relatively small volume of the tunnel in the vicinity of the UreD:(UreF)_2_ interface. Since we were not specifically interested in fragments, the docking outcome was then filtered to exclude such kinds of molecules. Moreover, in order to focus our attention on those molecules exploiting the entrance of the tunnel for establishing interactions, a measure of the buriedness was introduced (see Methods) that allowed us to exclude all the compounds that were only loosely bound to the external surface of UreD. The use of these filters narrowed down the top-ranking hitlist to a total number of 52 molecules that were then clustered to identify major scaffolds. In Table 2, the three top-scoring compounds binding this lateral-side site and belonging to distinct clusters, hereafter referred to as **L1–3**, are reported together with their ranking position in the original hitlist and some basic properties. Figure 3 complements Table 2 by showing the molecular structures of the compounds resulting from the virtual screening. As the table shows, the relatively low ranking assigned to these compounds reflects the fact that the top positions were occupied by fragment-like compounds. Furthermore, the unsatisfying docking scores showed by these compounds are an indication that F#2 might be a difficult site to target with molecule-sized scaffolds [56]. Interestingly, the docking poses for Compounds **L1–3** (Figure 4) show hydrophilic moieties, which are, in general, expected to be solvent-exposed, establishing polar interactions with the entrance of the tunnel. The fact that a putative tunnel for ion translocation is surrounded by hydrophilic residues is not surprising *per se*, and, to a certain extent, polar interactions with exogenous compounds are expected. On the other hand, the binding modes are somewhat flipped over, with the most polar sides within the site and the hydrophobic regions that are exposed to the solvent. (see Figure 4).

**Figure 5:**
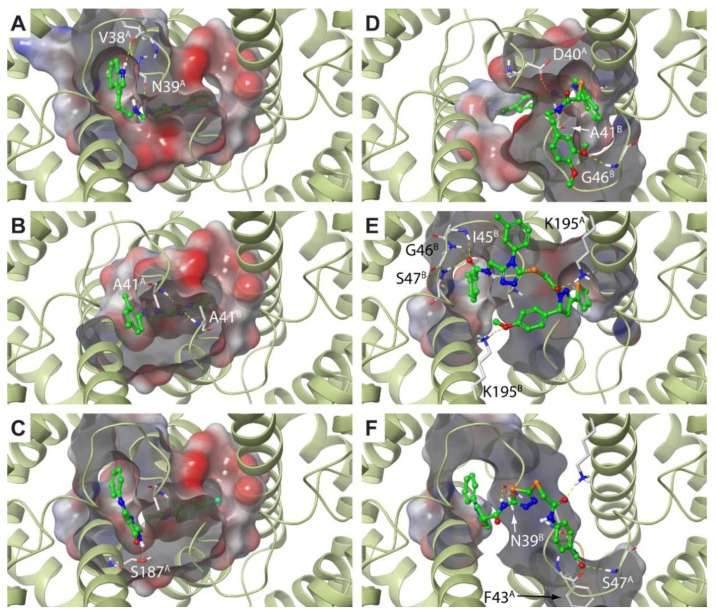
Docking poses for Compounds **B1–3** (Panels **A**–**C**) and **E1–3** (**D**–**F**) in the F#1 site. *Hp*(UreF)_2_ ribbons are in dark green, and residues involved in the binding are labeled and depicted as thin sticks colored according to atom type. Considering that the F#1 binding site is at the dimerization interface, the chain identifier has been added to the residue labels. The **B1–3** and **E1–3** compounds are shown as green sticks, where heteroatoms have been colored according to atom type. Hydrogen bonds are shown using yellow dashed lines.

The scenario is strikingly different in the case of the SBVS performed on the F#1 site. As shown in Table 2, the top-ranked non-fragment compound is located at Position 8 of the hitlist, showing a very favorable docking score of −10.37 (Compound **B1**). This is a clear indication that this site might be better suited for targeting by drugs [56]. Moreover, by visually inspecting the top-ranked hitlist, we observed an interesting behavior of the docking results. Specifically, we noticed that the binding mode adopted by these compounds could be ascribed to two major classes: buried and solvent-exposed (301 and 521 molecules, respectively, out of 1000). The buriedness descriptor turned out to be instrumental for unambiguously classifying molecules according to this feature (see Methods). In Table 2, the three top-ranked “buried” and “exposed” compounds (**B1–3** and **E1–3**, respectively) are reported and ranked according to their position in the original hitlist. As the table shows, all the compounds display very good docking scores, in striking contrast with the best candidates obtained for Site F#2. This is also reflected by the relatively high positions in the original hitlist for Compounds **B1–3** and **E1–3** (also see Figure 3). Concerning the distinct binding modes adopted by these compounds (Figure 5), they can be better understood by recalling the shape of the F#1 site and the fact that it is located at the origin of the main tunnels (see Figure 1D). As such, the screened compounds might bind the (UreF)_2_ dimer by either exploiting the (UreF)_2_:(UreG)_2_ interface as the main contact area or deeply inserting at the tunnel entrance (or both, if sufficiently large molecules are provided). We note that the shape and volume of the tunnels available for ligand binding are remarkably different in Sites F#1 and F#2. Indeed, while in the case of Site F#2, only fragments or small portions of molecules could be hosted, the tunnel entrance in Site F#1 is large enough to accommodate entire drug-like molecules (compare the buriedness values for the **L1–3** and **B1–3** series in Table 2). Conversely, the solvent-exposed molecules are only partly engaging the tunnel entrance, as they rather exploit the (UreF)_2_:(UreG)_2_ interface to establish favorable interactions (see Figure 4). As previously mentioned, the C_2_ symmetry of the binding site has a profound impact on the prioritized molecules, and not surprisingly, several solvent-exposed F#1 potential binders are, indeed, symmetric molecules. Even though Compounds **E1–3** cannot be strictly regarded as symmetric compounds, a pseudo-symmetric shape can still be recognized, with a central ring and two linkers in the case of **E1** and **E3** or a four-branched structure in the case of **E2**. These are recurring structural features that can be observed in many other compounds of this class. Symmetric binding sites are most often found in the case of ion channel cavities [57]. Here, we speculate that molecular symmetry can be a key molecular determinant for optimally exploiting the interactions with UreF at the (UreF)_2_:(UreG)_2_ interface.

### 2.3. Molecular Dynamics Simulations Confirm the High Druggability of Site F#1

In the previous section, we showed that Site F#1 is intrinsically better suited as a drug target site than F#2, not only in terms of shape and volume but also because Site F#1 allows greater variability in the potential binding modes. This variability can be virtually translated into design flexibility when it comes to identifying and optimizing specific binders. Still, for the sake of computational efficiency, docking scoring functions only provide very approximate estimates of drug–target affinities, especially those employed in the context of virtual screening [58]. Among the drawbacks of common docking programs and scoring functions, we mention that (i) strain energies and desolvation processes are disregarded, as only the bound state is explicitly considered in the calculation; (ii) the target is usually treated as a rigid body, so mutual relaxation upon binding is not described; and (iii) solvent effects are usually taken into account in a very approximate (and sometimes crude) way. All these approximations, that can be acceptable in the case of large library screenings, can potentially undermine the reliability of the identification of true binders. On top of that, force field-based scoring functions inherit all the approximations of the native force field, including difficulties in the representation of permanent electrostatics with atom-centered partial charges and the usual neglect of polarizability [59]. For these reasons, it is a common practice to validate docking programs for their ability to reproduce experimentally derived binding modes, and Virtual Screening protocols to statistically enrich the hitlist with known binders over non-binders and/or decoys over a random selection of molecules from the database [60]. In both cases, the validation relies on the availability of known molecules able to bind to the target of interest. Whenever a proper retrospect validation cannot be attained, it is always advisable to complement docking calculations with more rigorous computational approaches. A computationally expensive way is to refine the docking solutions with higher-level theory models, like Quantum Mechanics/Molecular Mechanics (QM/MM) methods [61,62]. Another widespread workaround is to combine the results of docking calculations with Molecular Dynamics (MD) simulations [63]. By doing so, the integrated procedure benefits from the speed of docking and the accuracy provided by MD-based approaches in refining and/or reranking the docking outcome. Indeed, with MD simulations, the protein–ligand system is modeled more realistically, including the explicit treatment of solvent molecules and allowing full flexibility for all the considered molecular entities. In particular, MD can be exploited to assess the reliability of binding modes from either a structural or energetic standpoint. Reranking binding poses through energetic rescoring is certainly the most elegant solution. This is generally achieved by relying on more or less elaborate thermodynamic cycles [64] or advanced simulative approaches [65], and it has been shown to significantly improve the SBVS performance [66]. Alternatively, one can assess the reliability of binding modes by evolving Newton’s equations of motion and comparing their relative stability, i.e., the ability to preserve the interactions identified through the docking procedure. Here, we followed both strategies, and we challenged the stability of the docking outcomes by performing the repeated MD simulation of the three top-ranked compounds for each binding site, F#1 and F#2. Moreover, in the case of F#1, both the buried and solvent-exposed binding solutions were considered. Each (UreF)_2_-ligand complex was simulated for 100 ns, and each simulation was repeated three times, totaling 2.7 μs of sampling as a whole. Then, the Molecular Mechanics Generalized Born Surface Area (MM-GBSA) method [67] was used to assess the binding free energy of the considered molecules. Specifically, the free energy was estimated using the VSGB 2.0 variant of the MM-GBSA scheme [68], and each contribution (see Methods) was computed in the form of ensemble averages by extracting evenly spaced configurations from the MD trajectory.

**Figure 6:**
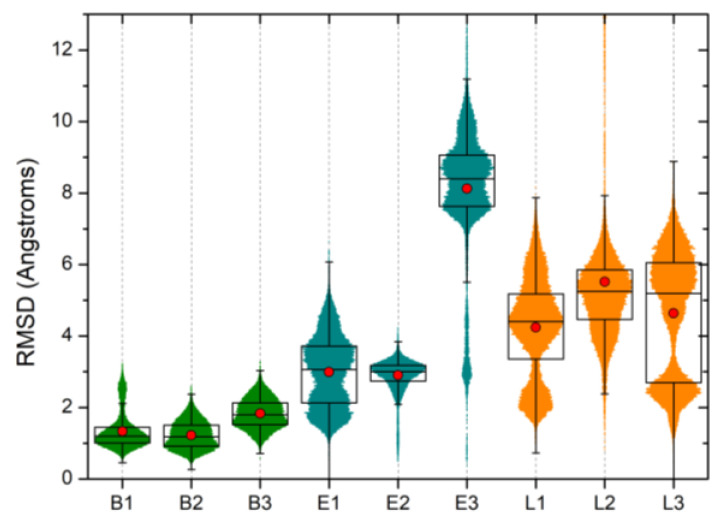
Violin plot of the root mean squared deviation (RMSD) calculated on the heavy atoms of each ligand with respect to the initial docking pose. For each ligand, the results of the three simulations were aggregated in order to increase the sampling. The width of each strip is proportional to the relative RMSD frequency. The average RMSD position is highlighted using a red dot. Each box represents the interval between the end of the first quartile and the beginning of the fourth one. The error bars represent the RMSD standard deviations. “Buried”, “Exposed”, and “Lateral” ligands are in green, cyan, and orange, respectively. See Figure S1 in the Supplementary Information for RMSD vs. simulation time plots.

In Figure 6, we report the root mean squared deviation (RMSD) calculated on the heavy atoms of each ligand with respect to the relaxed docking pose (i.e., the configuration corresponding to the energy-minimized and -thermalized pose before the MD production run) after optimal alignment on the Cα carbon atoms of the protein. The RMSD was calculated for every frame of the trajectory, and the results from the three independent runs of each (UreF)_2_-ligand complex were then aggregated. With the only exception of **E3** (see below), the RMSD distribution plots show a good correlation between the relative stability of compounds and the site druggability, as emerged from the previous analysis. Specifically, Compounds **L1–3** show significantly less stable binding modes compared to those binding to Site F#1. Concerning the relative stability of the buried and solvent-exposed molecules here considered as prototypical F#1 site binders, we note that **B1–3** show remarkably high stability, while **E1** and **E2** are somewhat halfway between the buried compounds and F#2 binders.

**Figure 7:**
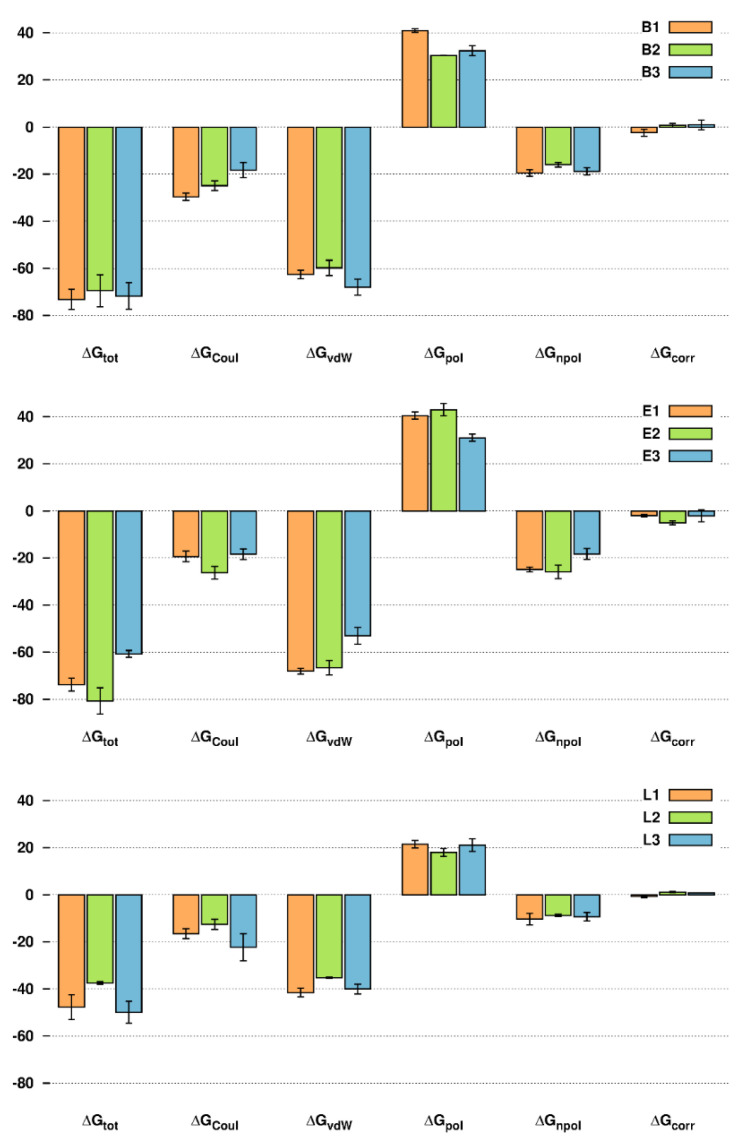
Results of the Molecular Mechanics Generalized Born Surface Area (MM-GBSA) analysis for Compounds **B1**–**3** (top panel), **E1**–**3** (central panel), and **L1**–**3** (bottom panel). The left bars report the total free energy of binding (ΔG_tot_). The electrostatic, van der Waals, polarization, nonpolar solvent–solute interaction, and total corrections contributions (ΔG_Coul_, ΔG_vdW_, ΔG_pol_, ΔG_npol_, and ΔG_corr_, respectively) are also reported.

A better description of the stability of binding modes can be obtained by complementing these results with clustering (Figures S2 and S3) and energetic analysis (Figure 7). Moreover, in Figures S4–S12 we also report a focus of the binding modes as identified by the docking program and the preservation of some key contacts in the representative structures of all the clusters identified along the MD trajectory. As expected, a remarkably small number of clusters was obtained for **B1–3** (no more than three clusters), with only one cluster significantly populated in the case of **B1** and **B2** (Figure S2). In the case of **B3**, the population of Cluster #1 is not as overwhelming as in the previous cases, but the contribution of the remaining clusters to the main binding mode is still marginal. Most importantly, the relaxed docking pose was always found in Cluster #1 for all the compounds of this class (see the red circle in Figure S3), in agreement with the pronounced thermal stability of the binding mode identified by virtual screening. As shown in Figures S4–S6, most of the main protein–ligand interactions found in the original docking pose are well preserved in all of the cluster representatives. This is also reflected by the particularly favorable binding energy obtained by MM-GBSA (−73.2, −69.5, and −71.7 kcal/mol for **B1**, **B2**, and **B3**, respectively). We stress that the absolute values obtained with this method are not expected to be physically relevant, and only comparisons among relative energies are meaningful. Moreover, entropic contributions were disregarded from the MM-GBSA computation, making comparisons between non-congeneric molecules rather arguable. Still, it is interesting to note that a similar energy was obtained for **E1** (−73.6 kcal/mol). Notably, even though a total of 35 clusters were identified for this molecule, only two of them turned out to be significantly populated (Figure S2), with the relaxed docking pose found in Cluster #2. By visually inspecting the representative structures (see Figure S2), we observed that the binding modes of Cluster #1 and #2 mostly differed by the orientation of the water-exposed dimethoxyphenyl group, explaining the relatively high RMSD value shown in Figure 6. Thus, the molecule was found to interconvert among two main metastable states that were almost equi-populated. As Figure S7 shows, all the interactions identified by the docking program were preserved in Cluster #2, as expected. However, it is interesting to note that in the representative member of Custer #1, only the Gly46A(N):O1 contact was lost, while the distance between the R_6_2 group and the aromatic moiety of Tyr48B was only slightly increased. This observation is a further indication that the variability in the main clusters of **E1** is of little relevance, as it mainly involves a solvent-exposed moiety of the molecule that mostly interacts with the same residues of the protein, possibly exploiting the intrinsic flexibility of the tyrosine’s sidechain. An interesting case is represented by **E2**, which shows an extremely narrow RMSD distribution peaking at about 3 Å away from the relaxed docking pose (Figure 6). Here, the MM-GBSA scheme returned the lowest energy among the considered molecules (−80.7 kcal/mol), while cluster analysis assigned the relaxed docking pose to Cluster #4 (out of 7). Since Cluster #1 has a relative population of >80%, and because a departure from the original binding mode was observed in all the replicates of the MD simulations (see Figure S1), we can conclude that this molecule is potentially able to effectively interact with the F#1 site with a binding mode that slightly differs from the one originally proposed by the SBVS. This is confirmed by Figure S8, showing that two polar contacts (the charge-assisted hydrogen bonds between Lys195A/B(NZ):O2/O3) found in the bare docking pose were already lost upon energy minimization and thermalization (Cluster #4 in the same Figure). This is not surprising, as solvent-exposed ionic interactions and charge-assisted hydrogen bonds are known to be much less relevant than those established deep within binding pockets, and it represents a typical example highlighting the importance of relaxing the docking solution through subsequent MD simulations. Another striking rearrangement is found in the case of **E3**, but this time, the molecule seems unable to find a favorable binding mode during the timescales of the MD simulations. Indeed, the analysis of the trajectories suggests the observation of the initial steps of an incipient unbinding process rather than a simple interconversion towards a different metastable state. This behavior is supported by the relatively low binding energy (−61.3 kcal/mol) and the high number of clusters identified (82, with the relaxed docking pose found in Cluster #28). More specifically, Figure S9 shows that all the leading polar interactions found at the beginning of the MD simulations were lost in most of the clusters (compare, for instance, Cluster #1 to Cluster #28), while the apolar contacts were at least partly retained (but not always, e.g., Cluster #10). We can speculate that the disagreement between the docking outcome and the MD behavior can be attributed to the adoption of a highly bent conformation of the ligand that, even though it can be predicted as a local minimum from the static docking program, quickly relaxes to a lesser strained conformation as soon as MD simulation is performed.

Concerning the class of molecules binding Site F#2, the MM-GBSA analysis confirmed their less favorable binding energy compared to that of the previous ones, in line with the RMSD distribution plot shown in Figure 6. In particular, binding energies of −47.7 and −49.9 kcal/mol were obtained for **L1** and **L3**, respectively, while a much higher value was found for **L2** (−37.5 kcal/mol), for which a full unbinding event was recorded in one of the three runs (see Figure S1). The cluster analysis is consistent with this trend, showing total numbers of clusters of 35, 127, and 42 for **L1**, **L2**, and **L3**, respectively (Figure S3). The analysis of contacts shown in Figures S10–S12 confirms the fact that these molecules bind the entrance of the tunnel with their polar moiety, and, at least for **L1** and **L3**, these interactions are preserved in most of the clusters. Conversely, the interactions established with the apolar residues at the UreD:(UreF)_2_ interface involve the more solvent-exposed portion of these compounds (aromatic rings). Probably, because of the unsuitable shape of the interface, these latter contacts seem not to be properly optimized, and this is clearly represented by the absence of key apolar interactions in most of the cluster members in Figures S10–S12. Taken as a whole, these results highlight the fact that these molecules are less suited as potential binders of the F#2 site, as already predicted by the low values of their docking scores (Table 2). While only **L2** left the binding site during the timescales of our repeated MD simulations, it is possible to speculate that, with more and possibly longer MD runs, this is a behavior that might be also observed for other compounds of this series. In spite of this, it is also likely that the small size and the specific chemical nature of the polar moiety of **L2** bound into the tunnel might have accelerated this behavior over **L1** and **L3**. Even though much more accurate computational approaches are required to unambiguously assess the relative stability of the obtained binding modes, we believe that this does not significantly affect our interpretation of the relative druggability of Sites F#1 and F#2. Indeed, one has to bear in mind that only three molecules were considered as representative of each site/binding mode, while a certain amount of individual variability is expected. In other words, it is reasonable to think that the structural determinants of some specific molecules might override the overall features displayed by the sites. Besides, an MD refinement of the entire hitlist would be needed to assess, in a statistically robust way, the relative propensity of the sites to be exploited for drug discovery, but this is clearly out of reach with conventional computational resources.

Having ruled out Site F#2, what are the potential mechanistic implications of targeting Site F#1 with buried or solvent-exposed binders? When looking for PPI disruptors, one is usually concerned with molecules able to bind the surface of one of the two molecular partners involved at the protein–protein interface [69,70]. Even though exceptions to this general strategy are known [71], this is the mechanism by which solvent-exposed F#1 binders are expected to function. Conversely, buried F#1 binders are located deep within the internal cavity of the (UreF)_2_ dimer, and because of that, they are not necessarily able to reach the (UreF)_2_:(UreG)_2_ interface, where the disrupting effect would take place (see Figure S13). Nonetheless, advocating a different mechanism, we speculate that buried F#1 binders might still act as effective indirect urease inhibitors. Indeed, by reducing tunnel accessibility through steric hindrance, this class of molecules would act as blockers of Ni(II) translocation, which is an essential step for the maturation of the urease enzyme.

## 3. Materials and Methods

### 3.1. Structure-Based Virtual Screening

The *Hp*UreDFG complex was retrieved from the Protein Data Bank (PDB id 4HI0) [49] and then decomposed into its main components, namely the two *Hp*UreD monomers and the *Hp*(UreF)_2_ and *Hp*(UreG)_2_ dimers. The putative binding sites were detected by running SiteMap [53,54] (using default parameters), available in the Schrödinger suite 2016-3 (Schrödinger, LLC, New York, NY, USA, 2016). The *Hp*UreD monomer and the *Hp*(UreF)_2_ dimer were used as targets, while the *Hp*(UreG)_2_ dimer was not further considered because this is protein is supposed to be located at the beginning of the tunnels required for the transport of Ni(II) ions and because of the unfavorable shape of the protein surface at the interface with the *Hp*(UreF)_2_ dimer. Five binding sites were identified for each protein (see Table 1). The two top-ranked sites of the *Hp*(UreF)_2_ dimer were used to perform the virtual screening (Site F#1 and Site F#2 in Table 1). The *Hp*(UreF)_2_ dimer was then processed with the Schrodinger “Protein Preparation Wizard” tool [72]. The H-bonds were assigned with PROPKA 3 at pH 7.0, and a restrained minimization was performed to relieve steric clashes using a convergence criterion of 0.30 Å of the root-mean-square deviation (RMSD) for the heavy atoms with respect to the initial structure and OPLS3 [73] as a force field.

The molecular database was prepared with the Schrodinger suite, starting from 2D structures taken from the ZINC database (www.zinc.docking.org) [55], including Asinex, Chembridge, Princeton, NCI, and ZINC natural as vendors. The 2D structures were converted into 3D structures, and stereoisomers were generated at pH 7.0 ± 1.0 with the tools Ligprep and Epik of the Schrodinger suite using OPLS3 [73] as a force field. The Qikprop utility (Schrödinger) was used to compute the “absorption, distribution, metabolism, elimination and toxicity” (ADMET) properties for each compound of the database and to filter them according to a “soft Lipinsky rule” (Molecular weight (MW) ≤ 600, Rotatable bonds ≤ 10, Number of H-bond acceptors ≤ 10, Number of H-bond donors ≤ 5, Number of chiral centers ≤ 2, QplogPo/w ≤ 6).

Two runs of virtual screening were performed by centering the grid on the aforementioned sites on the surface of the *Hp*(UreF)_2_ dimer. The database was docked using the standard precision (SP) scoring function of the Glide software (Schrödinger) [74,75,76], and, for each run, the top-ranking 1000 molecules were selected. To better analyze the docked molecules, a filter based on the MW and the relative buriedness (*B*), which we defined as

(1) $$B=1-\left(\frac{SASA_{\mathrm{bound}}}{SASA_{\mathrm{unbound}}}\right),$$

were used, where *SASA*_bound_ and *SASA*_bound_ are the solvent accessible surface area of the ligand in the bound and unbound state, respectively, calculated with Schrödinger. According to Equation (1), an unbound molecule will have *B* = 0, while a completely buried molecule will have *B* = 1. In order to retrieve only the larger molecules that were effectively bound to the lateral-side site, the compounds were filtered according to the criteria *MW* ≥ 300 and *B* ≥ 0.75. Conversely, by visually inspecting the molecules located on the top-side site, we noticed that a fraction of them was able to enter the cavity in a more effective way than the remaining one. To classify the top-side site docking outcome based on this behavior, the compounds were grouped as “buried” or “exposed” based on the criteria (*MW* ≥ 300 and *B* ≥ 0.90) and (*MW* ≥ 300 and 0.80 ≤ *B* < 0.90), respectively. The filtered compounds were then clustered using the Tanimoto distance as a metric based on the Molprint2D (64-bit) fingerprints with CANVAS [77,78]. After visual inspection, the three best-scoring molecules belonging to distinct clusters were selected for each group (“lateral”, “buried”, and “exposed”).

### 3.2. Molecular Dynamics

All simulations were performed through the Desmond 5.9 molecular dynamics software [79] running under Schrödinger and using the OPLS3 force field [73]. The simulation parameters and protocols are summarized in Table 3.

In order to improve the statistics in the following analysis, the three trajectories done for each molecule were combined. The RMSD was calculated by superimposing the protein backbone and calculated on the ligand heavy atoms. Cluster analysis was performed on the heavy atoms of the ligands using the cluster tool included in Gromacs 2016.4 [80,81,82] and the gromos algorithm [83]. A 2.0 Å cut-off for the RMSD was used to include structures in the same cluster.

### 3.3. MM-GBSA Analysis

Molecular Mechanics Generalized Born Surface Area (MM-GBSA) [67] was applied to calculate the binding free energy of the complexes. In particular, the VSGB 2.0 model was used [68]. The latter includes an optimized implicit solvent model as well as physics-based corrections for hydrogen bonding, π–π, self-contact, and hydrophobic interactions. The VSGB 2.0 energy function (Equation (2)) contains the OPLS-AA protein force field bonded and nonbonded terms [84], summed to a solvation term and the above-reported correction terms.

(2) $$G_{total}=E_{\mathrm{OPLS}-\mathrm{AA}}+G_{sol}+\sum E_{corrections}$$

In a typical Generalized Born model, the solvation free energy (*G_sol_*) is expressed as the sum of a cavity term (*G_cav_*), a van der Waals term (*G_vdW_*), and a polarization term (*G_pol_*) (Equation (3)).

(3) $$G_{sol}=G_{cav}+G_{vdW}+G_{pol}$$

The nonpolar solvent–solute interaction is usually calculated by summing the cavity and the van der Waals terms. In the VSGB 2.0 approach, the latter sum is determined by using a parameterized hydrophobic term. The polar solvent–solute interaction is represented by the polarization term (Equation (4)), which depends on the solvent and internal dielectric constants, the partial charges, and a function (*f_GB_*) of the distances between two atoms (*r_ij_*) and their generalized Born radii (*α_i_* and *α_j_*) (Equation (5)).

(4) $$G_{pol}=-\frac{1}{2}\left(\frac{1}{\varepsilon_{in\left(ij\right)}}-\frac{1}{\varepsilon_{sol}}\right)\underset{i<j}{\sum }\frac{q_{i}q_{j}}{f_{GB}}$$

where the internal dielectric constant *ε_in_*_(*ij*)_ can vary from 1.0 to 4.0 as the maximum value of the internal dielectric constants of atom *i* and atom *j*. *ε_sol_* is the solvent dielectric constant.

(5) $$\begin{array}{c} f_{GB}=\sqrt{r_{ij}^{2}+\alpha_{ij}^{2}e^{-D}}\\ \alpha_{ij}=\sqrt{\alpha_{i}\alpha_{j}}\\ D=\frac{r_{ij}^{2}}{{\left(2\alpha_{ij}\right)}^{2}} \end{array}$$

The physics-based correction terms (*E_corrections_*) include (i) the use of an empirical functional form to enforce hydrogen bond angles and distances; (ii) an explicit π–π packing correction for pairs of amino acid side chains including a conventional aromatic moiety (Phe, Tyr, His, and Trp) as well as Y-aromatic structures (Arg, Asn, and Gln); (iii) a term dealing with the side chains of Asn, Gln, Ser, and Thr interacting with their own backbone nitrogen or oxygen atoms; and (iv) a hydrophobic term rewarding contacts between nonpolar heavy atoms and stabilizing hydrophobic contacts [68].

The MM-GBSA analysis was performed with the *thermal_mmgbsa.py* script from the Prime module [85,86], available in the Schrödinger suite, and averaging over 500 configurations evenly extracted from the MD trajectories.

## 4. Conclusions

In this work, we explored the feasibility of using Structure-Based Virtual Screening to identify potential molecules able to interfere with the functionality of the (*Hp*UreDFG)_2_ complex. This molecular assembly represents an accessory complex that is required for the activation of the urease enzyme; therefore, the molecules identified through the *in silico* screening can be regarded as potential indirect urease inhibitors. MD simulations turned out to be instrumental for refining the docking outcome. Moreover, they helped in assessing the relative stability of binding modes and the reliability of putative sites on the (UreF)_2_ surface, where future computational and experimental drug discovery campaigns should be directed. Finally, we showed that potential indirect urease inhibitors targeting the (UreF)_2_ dimer can exert their activity by either functioning as classical protein–protein disruptors or physically blocking Ni(II) transfer, which is essential for urease activation. To the best of our knowledge, this is the first report of small molecules specifically intended as tunnel blockers. Even though we are aware that further calculations, as well as experimental assays, are necessary to corroborate these theoretical findings, the results presented in this work could add to the body of knowledge on this topic and inform future studies in this direction.

## Figures and Tables

**Table 1:** Ranking of druggable sites at the surface of the *Hp*UreD monomer and the *Hp*(UreF)_2_ dimer.

Ranking	SiteScore	DrugScore	Volume (Å^3^)
***Hp*** **UreD**			
D#1	1.004	0.986	167.770
D#2	0.800	0.778	90.766
D#3	0.785	0.792	144.060
D#4	0.610	0.546	91.924
D#5	0.610	0.514	71.644
***Hp*** **UreF**			
F#1	1.087	1.103	643.554
F#2	0.849	0.818	109.031
F#3	0.738	0.700	125.538
F#4	0.864	0.778	108.645
F#5	0.942	0.700	107.616

**Table 2:** Prioritized molecules obtained from the structure-based virtual screening (SBVS) performed on Sites F#1 and F#2. Molar weight and log*p* values were taken from the ZINC database entries [55].

Name	ZINC Id	Ranking	Docking Score	Cluster Id.	Log*p*	MW (Da)	Buriedness
**F#2**							
L1	ZINC9827332	314	−6.38	4	−0.852	334.401	0.798
L2	ZINC211138067	361	−6.33	2	2.404	301.371	0.776
L3	ZINC4940493	494	−6.22	1	0.841	341.371	0.779
**F#1**							
B1	ZINC4373981	8	−10.37	1	2.954	380.423	0.992
E1	ZINC97963747	11	−10.31	3	5.193	552.096	0.870
B2	ZINC13707391	21	−10.15	3	3.572	336.395	0.983
B3	ZINC9507588	24	−10.09	5	3.858	445.426	0.984
E2	ZINC97961716	32	−10.00	5	6.354	636.803	0.839
E3	ZINC9517885	36	−9.92	2	2.359	496.574	0.813

**Table 3:** MD simulation parameters and protocols used in this study.

**Statistical Ensemble**	NPT
**Production time**	100 ns
**Number of repeated runs per complex**	3
**Timestep: bonded, near, far**	2 fs, 2 fs, 6 fs
**Cutoff short-range interactions**	8.0 Å
**Thermostat**	Langevin, relaxation time 1.0 ps
**Temperature**	300 K
**Barostat**	Langevin, relaxation time 2.0 ps
**Pressure**	1 atm
**Heating and equilibration protocol**	100 ps, T = 10 K, Brownian dynamics NVT, solute heavy atoms restrained
	12 ps, T = 10 K, MD NVT, solute heavy atoms restrained
	12 ps, T = 10 K, MD NPT, solute heavy atoms restrained
	12 ps, T = 300 K, MD NPT, solute heavy atoms restrained
	24 ps, T = 300 K, MD NPT, no restraints
**Hardware**	NVIDIA GTX980

## Supplementary Materials

Supplementary Materials are available online. Figure S1: Plots of the RMSD calculated on the heavy atoms of each ligand with respect to the initial docking pose *vs.* simulation time. For each ligand, the three simulations are shown in black, red, and blue; Figure S2. Relative population of the ten most populated clusters obtained from the cluster analysis of the MD trajectories of compounds **B1–3** (left panels) and **E1–3** (right panels). The red dots highlight the cluster containing also the relaxed docked pose. In the insets is reported the detail of the representative structure for the most populated clusters of each compound. The starting docked pose is in white, while the pose representative of the cluster is in the same color of the bar in the plot; Figure S3: Relative population of the ten most populated clusters obtained from the cluster analysis of the MD trajectories of compounds **L1–3**. The red dots highlight the cluster containing also the relaxed docked pose. In the insets is reported the detail of the representative structure for the most populated clusters of each compound. The starting docked pose is in white, while the pose representative of the cluster is in the same color of the bar in the plot; Figures S4–S12: Comparison of the binding mode predicted by the docking program (top) and the preservation of key interactions in the clusters identified along the MD trajectory (bottom). Polar and apolar interactions are labeled in blue and gray, respectively; Figure S13: Longitudinal section of the solvent-excluded surface of the apo *Hp*UreDFG crystal structure (PDB id 4HI0) with an overlay of docking solutions for compounds **B1–3**. *Hp*UreD, *Hp*UreF, and *Hp*UreG chains are colored as in Figure 1A. Compounds **B1–3** are reported as “spheres” colored accordingly to the atom type.
