# Supplementary material for: Targeting the Protein Tunnels of the Urease Accessory Complex: A Theoretical Investigation
Source: Molecules. 2020 Jun 24;25(12):2911. doi: 10.3390/molecules25122911 (PMC7355429; doi:10.3390/molecules25122911)
Supplement: Supplementary file 1 [file molecules-25-02911-s001.pdf]

Targeting the protein tunnels of the urease accessory complex: a theoretical investigation

Matteo Masetti [1,¥], Federico Falchi [1,2,¥], Dario Gioia [3], Maurizio Recanatini [1], Stefano Ciurli [4] and Francesco Musiani [4,*]

[1] Laboratory of Computational Medicinal Chemistry, Department of Pharmacy and Biotechnology, University of Bologna, Bologna, Italy; matteo.masetti4@unibo.it, maurizio.recanatini@unibo.it
[2] Present affiliation: Molecular Horizon srl, Bettona (PG), Italy; federico.falchi@hotmail.com
[3] Computational and Chemical Biology, Italian Institute of Technology (IIT), Genova, Italy; dario.gioia@iit.it
[4] Laboratory of Bioinorganic Chemistry, Department of Pharmacy and Biotechnology, University of Bologna, Bologna, Italy; stefano.ciurli@unibo.it
[¥] M.M. and F.F. contributed equally.
[*] Correspondence: francesco.musiani@unibo.it; Tel.: +39 051 2096236

Supplementary Information

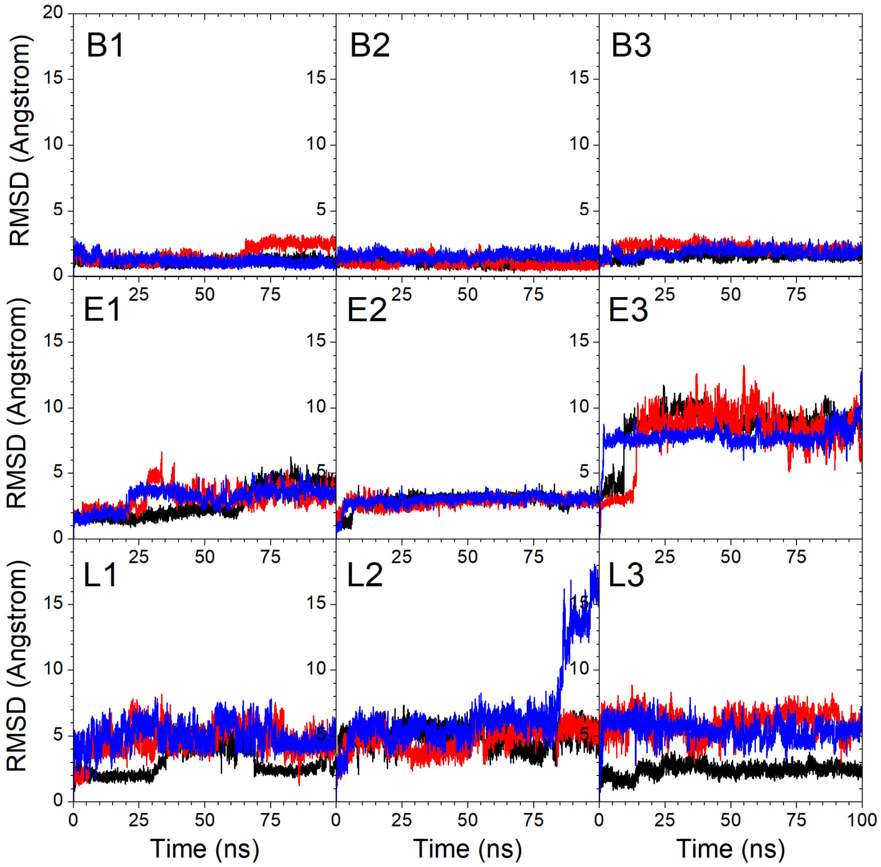

Figure S1. Plots of the RMSD calculated on the heavy atoms of each ligand with respect to the relaxed docking pose *vs.* simulation time. For each ligand, the three simulations are shown in black, red, and blue.

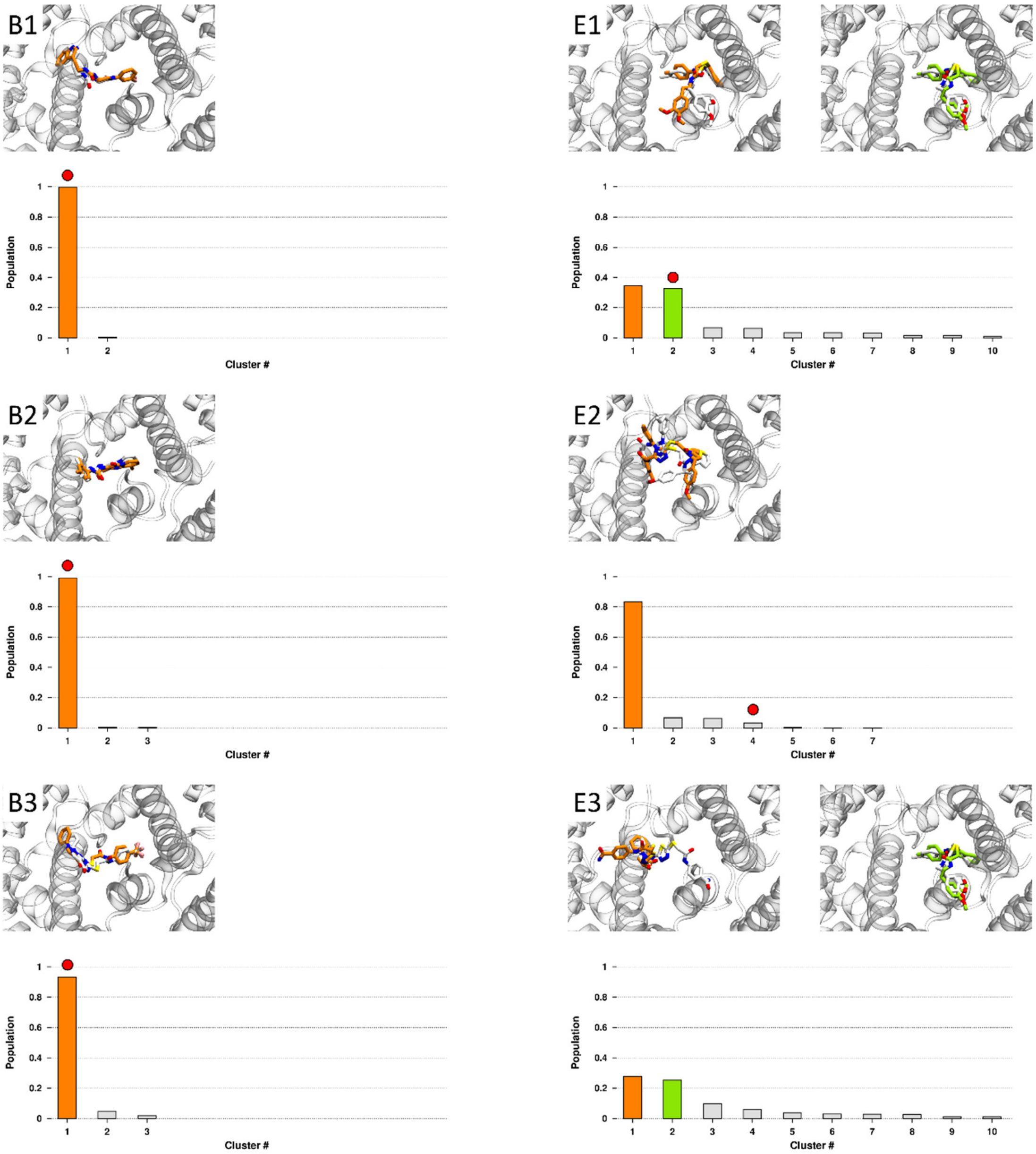

Figure S2. Relative population of the ten most populated clusters obtained from the cluster analysis of the MD trajectories of compounds **B1-3** (left panels) and **E1-3** (right panels). The red dots highlight the cluster containing also the relaxed docked pose. In the insets is reported the detail of the representative structure for the most populated clusters of each compound. The starting docked pose is in white, while the pose representative of the cluster is in the same color of the bar in the plot.

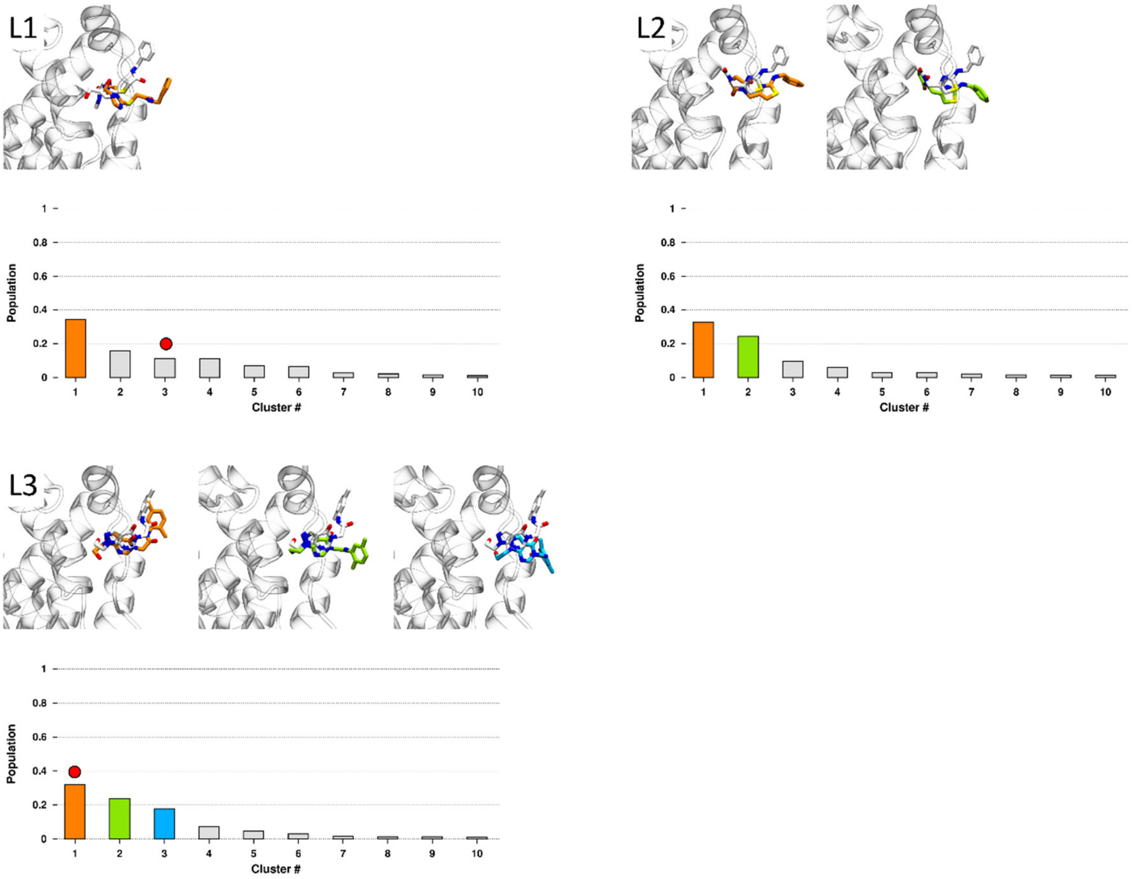

Figure S3. Relative population of the ten most populated clusters obtained from the cluster analysis of the MD trajectories of compounds **L1-3**. The red dots highlight the cluster containing also the relaxed docked pose. In the insets is reported the detail of the representative structure for the most populated clusters of each compound. The starting docked pose is in white, while the pose representative of the cluster is in the same color of the bar in the plot.

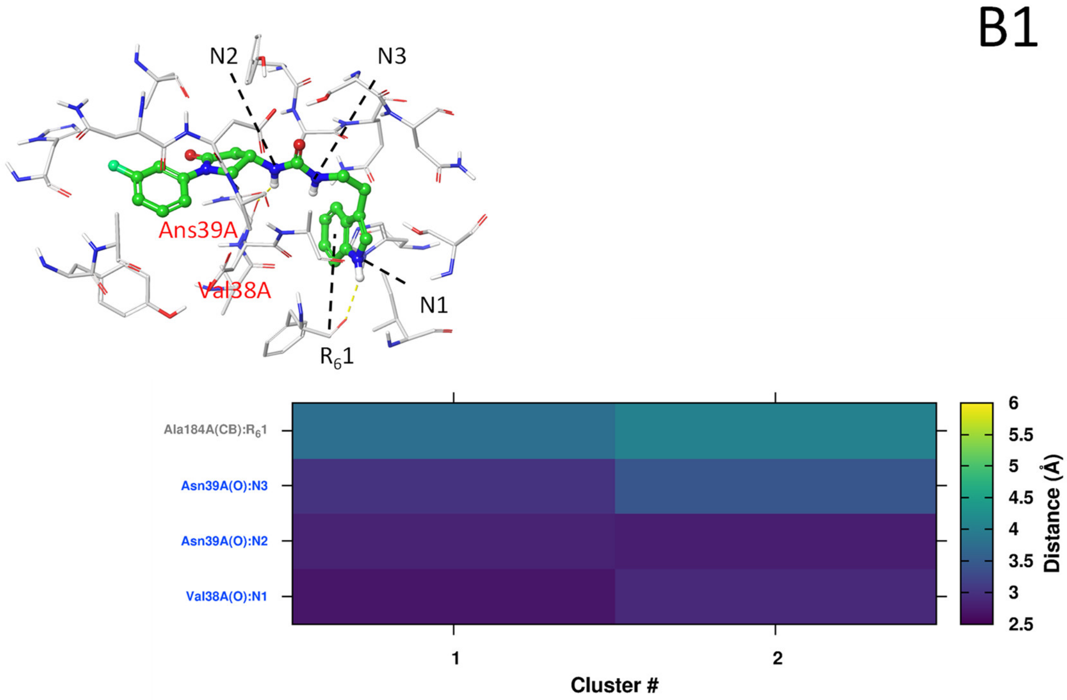

Figure S4. Comparison of the binding mode predicted by the docking program (top) and the preservation of key interactions in the clusters identified along the MD trajectory (bottom). Polar and apolar interactions are labeled in blue and gray, respectively.

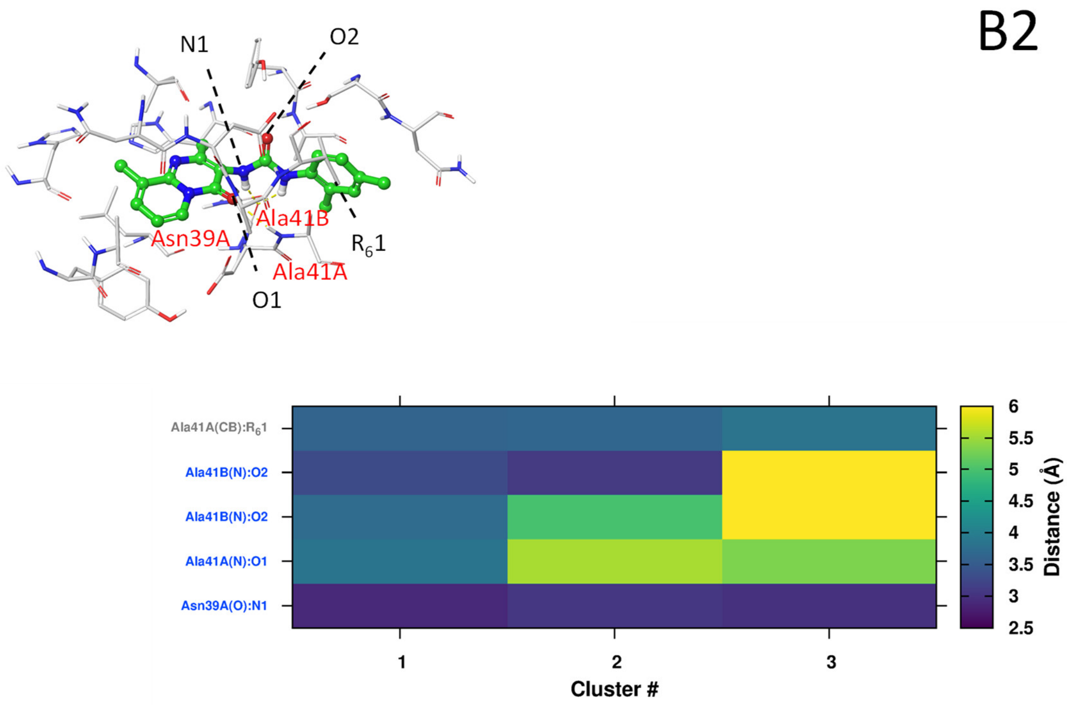

Figure S5. Comparison of the binding mode predicted by the docking program (top) and the preservation of key interactions in the clusters identified along the MD trajectory (bottom). Polar and apolar interactions are labeled in blue and gray, respectively.

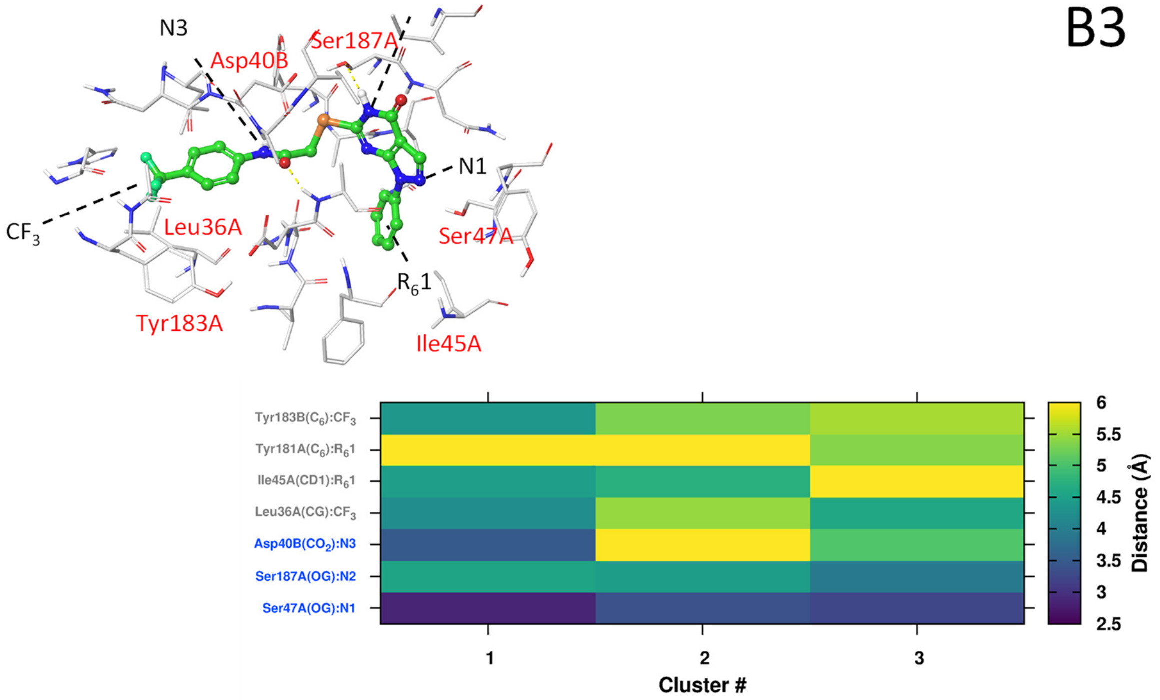

Figure S6. Comparison of the binding mode predicted by the docking program (top) and the preservation of key interactions in the clusters identified along the MD trajectory (bottom). Polar and apolar interactions are labeled in blue and gray, respectively.

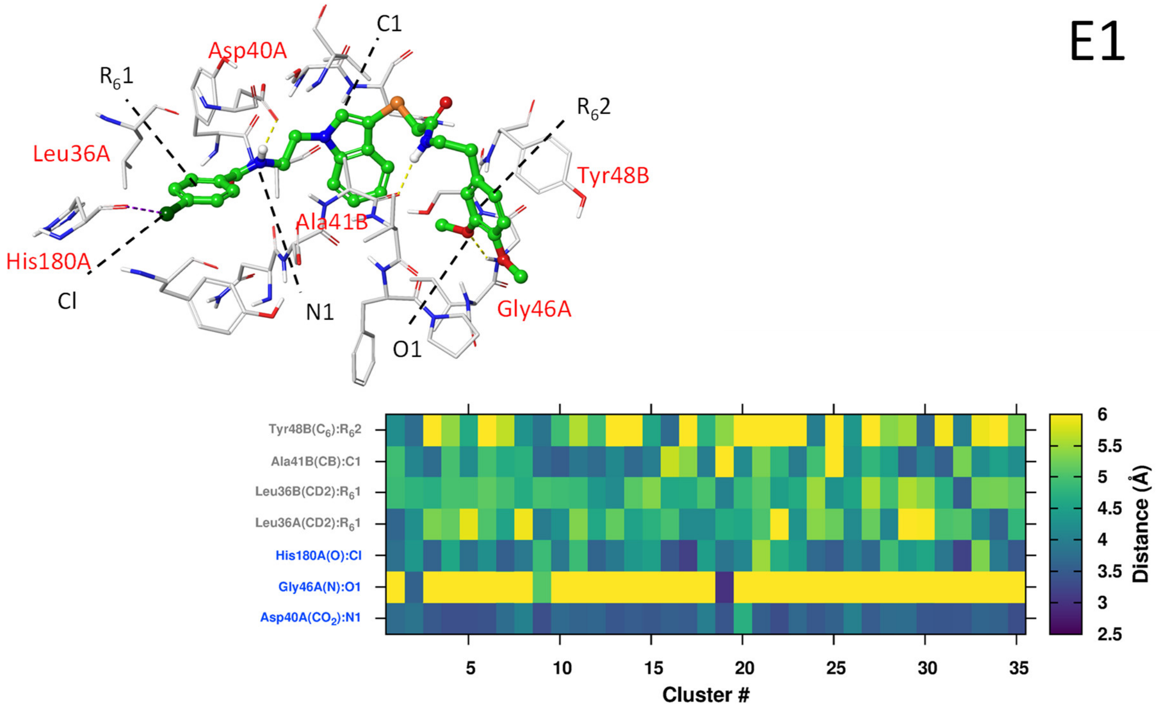

Figure S7. Comparison of the binding mode predicted by the docking program (top) and the preservation of key interactions in the clusters identified along the MD trajectory (bottom). Polar and apolar interactions are labeled in blue and gray, respectively.

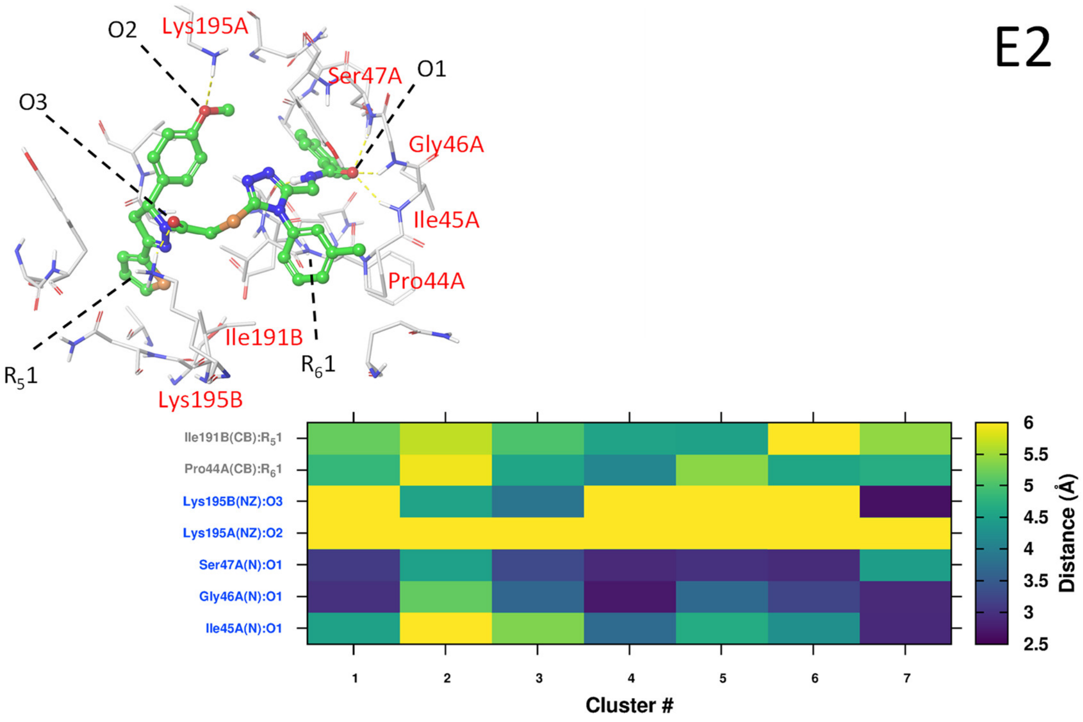

Figure S8. Comparison of the binding mode predicted by the docking program (top) and the preservation of key interactions in the clusters identified along the MD trajectory (bottom). Polar and apolar interactions are labeled in blue and gray, respectively.

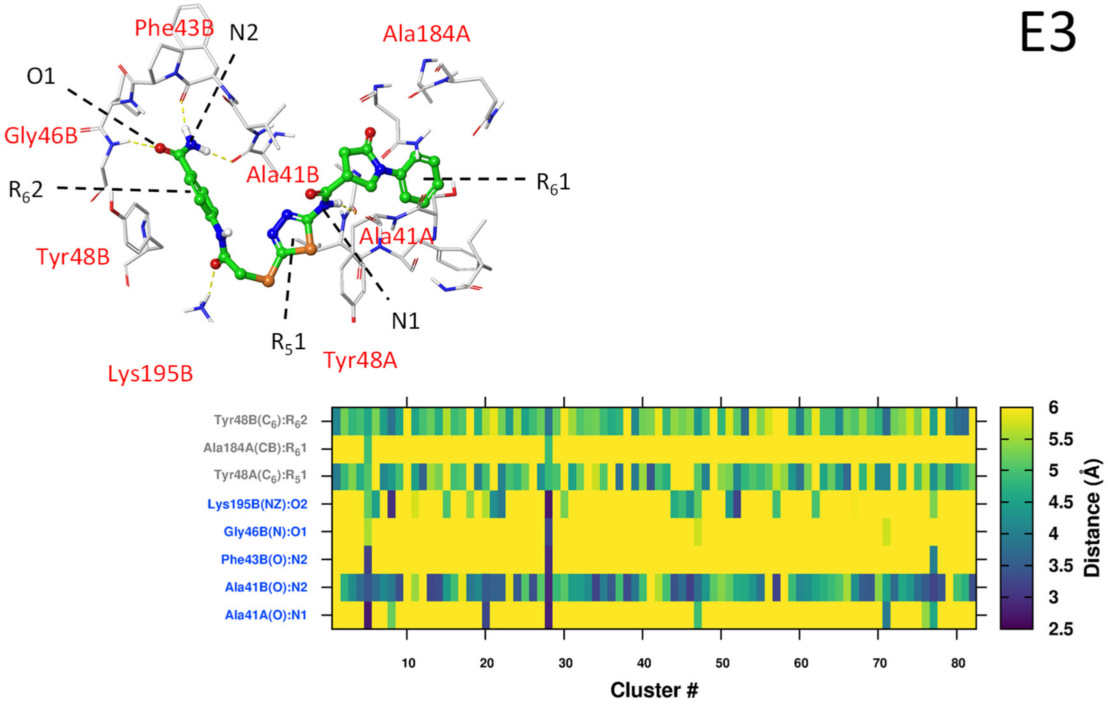

Figure S9. Comparison of the binding mode predicted by the docking program (top) and the preservation of key interactions in the clusters identified along the MD trajectory (bottom). Polar and apolar interactions are labeled in blue and gray, respectively.

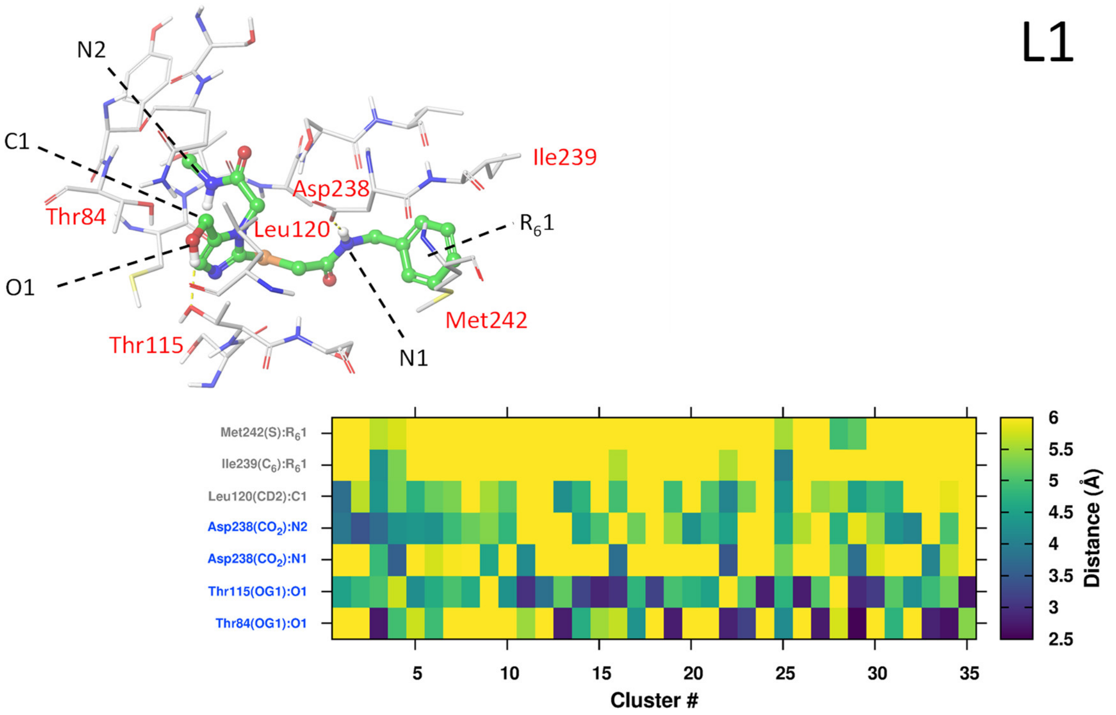

Figure S10. Comparison of the binding mode predicted by the docking program (top) and the preservation of key interactions in the clusters identified along the MD trajectory (bottom). Polar and apolar interactions are labeled in blue and gray, respectively.

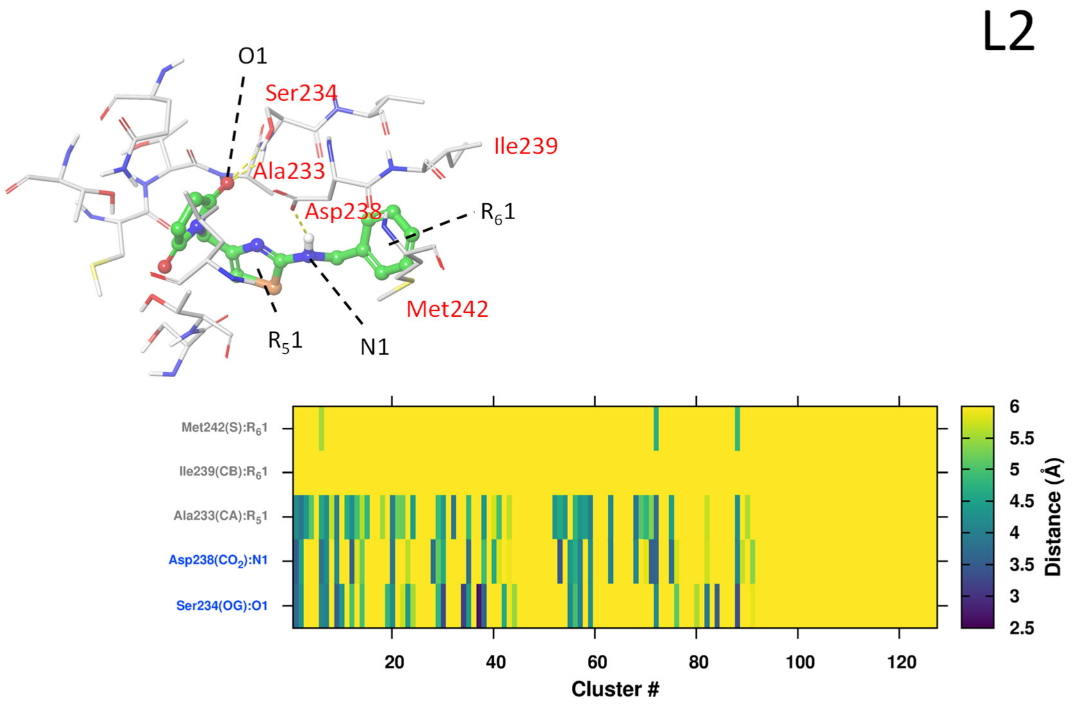

Figure S11. Comparison of the binding mode predicted by the docking program (top) and the preservation of key interactions in the clusters identified along the MD trajectory (bottom). Polar and apolar interactions are labeled in blue and gray, respectively.

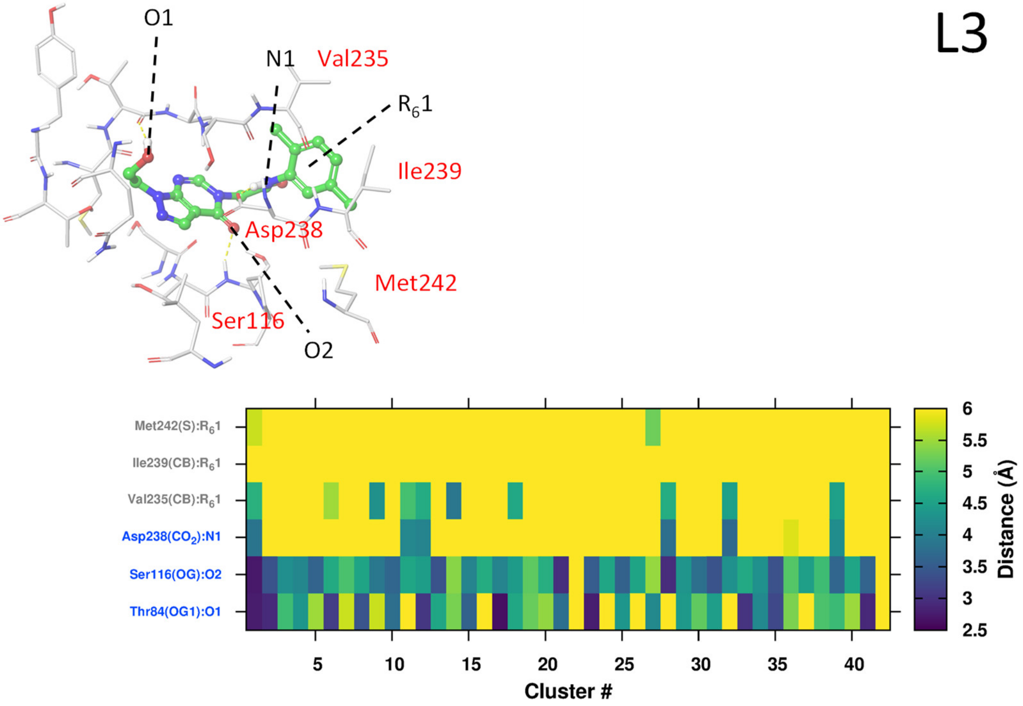

Figure S12. Comparison of the binding mode predicted by the docking program (top) and the preservation of key interactions in the clusters identified along the MD trajectory (bottom). Polar and apolar interactions are labeled in blue and gray, respectively.

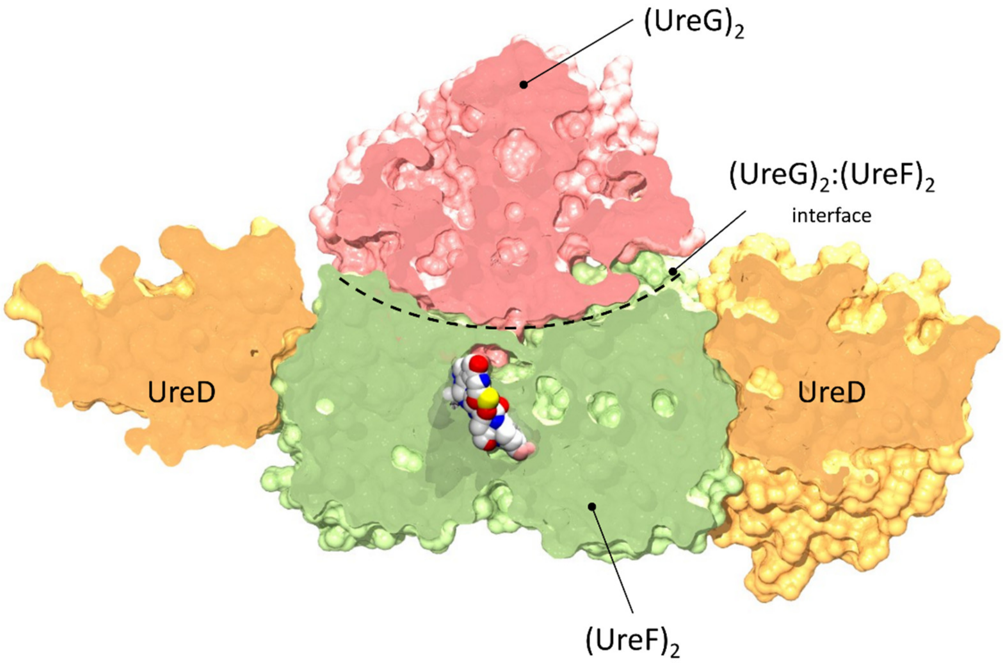

Figure S13. Longitudinal section of the solvent-excluded surface of the apo *Hp*UreDFG crystal structure (PDB id 4HI0) with an overlay of docking solutions for compounds **B1-3**. *Hp*UreD, *Hp*UreF, and *Hp*UreG chains are colored as in Figure 1A. Compounds **B1-3** are reported as “spheres” colored accordingly to the atom type.
